# Changes in Iron Metabolism and Oxidative Status in STZ-Induced Diabetic Rats Treated with Bis(maltolato) Oxovanadium (IV) as an Antidiabetic Agent

**DOI:** 10.1155/2014/706074

**Published:** 2014-01-05

**Authors:** Cristina Sánchez-González, Carlos López-Chaves, Cristina E. Trenzado, Pilar Aranda, María López-Jurado, Jorge Gómez-Aracena, María Montes-Bayón, Alfredo Sanz-Medel, Juan Llopis

**Affiliations:** ^1^Department of Physiology, Faculty of Pharmacy, University of Granada, 18071 Granada, Spain; ^2^Department of Cellular Biology, Faculty of Sciences, University of Granada, 18071 Granada, Spain; ^3^Department of Analytical Chemistry, Faculty of Chemistry, University of Oviedo, 33007 Oviedo, Spain; ^4^Department of Preventive Medicine and Public Health, Faculty of Medicine, University of Málaga, 29071 Málaga, Spain

## Abstract

The role of vanadium as a micronutrient and hypoglycaemic agent has yet to be fully clarified. The present study was undertaken to investigate changes in the metabolism of iron and in antioxidant defences of diabetic STZ rats following treatment with vanadium. Four groups were examined: control; diabetic; diabetic treated with 1 mgV/day; and Diabetic treated with 3 mgV/day. The vanadium was supplied in drinking water as bis(maltolato) oxovanadium (IV) (BMOV). The experiment had a duration of five weeks. Iron was measured in food, faeces, urine, serum, muscle, kidney, liver, spleen, and femur. Superoxide dismutase, catalase, NAD(P)H: quinone-oxidoreductase-1 (NQO1) activity, and protein carbonyl group levels in the liver were determined. In the diabetic rats, higher levels of Fe absorbed, Fe content in kidney, muscle, and femur, and NQO1 activity were recorded, together with decreased catalase activity, in comparison with the control rats. In the rats treated with 3 mgV/day, there was a significant decrease in fasting glycaemia, Fe content in the liver, spleen, and heart, catalase activity, and levels of protein carbonyl groups in comparison with the diabetic group. In conclusion BMOV was a dose-dependent hypoglycaemic agent. Treatment with 3 mgV/day provoked increased Fe deposits in the tissues, which promoted a protein oxidative damage in the liver.

## 1. Background

Diabetes is a pathological state that affects many metabolic processes and is associated with alterations to antioxidant defences and the metabolism of trace elements, which may contribute to the development of the disease.

The role of vanadium (V) as a micronutrient for humans, its essentiality, distribution, and toxicology, as well as its biological and pharmacological activity, are still not fully understood [1]. Rising levels of V in the environment, together with growing interest in the pharmacological effects of some of its compounds, have led to the study of V metabolism becoming an important area of current research.

In plasma, V is bound mainly to transferrin and to a lesser degree to other molecules, such as albumin [2–4]. Elsewhere, it is distributed primarily in the bone, kidney, and liver [1, 2, 4]. However, its interactions with other elements are not well understood.

Some V complexes have been shown to possess hypoglycaemic or insulin-mimetic properties [5–8], but many aspects remain to be determined, such as the interactions with other elements involved in antioxidant defence. Moreover, weight training athletes are reported to use it to improve performance [9]. According to some authors, V improves glucose metabolism and prevents the oxidant damage caused by diabetes [10].

However, other authors have related V with prooxidant effects [11, 12], because it does not normalize alterations in the metabolism of various elements that are associated with diabetes [12]. Therefore, the widespread use of V as a potential insulin-mimetic agent is subject to evaluation of the toxicity associated with its prooxidant effect.

Diabetes has been associated with alterations in iron (Fe) homeostasis both in humans and in animal models [13]. The generation of reactive oxygen species by prooxidant metals such as Fe may be a mechanism which aggravates this disease. Previous studies have reported that diabetes produces an accumulation of Fe in the tissues together with increased excretion of this metal [14]. Concerning the effect of diabetes on Fe content in the liver, opinions are mixed; some authors have observed increased Fe [15], while others have found no changes [11]. Little is known about the effects of exposure to V on Fe metabolism in diabetes [11] and therefore we consider it important to study whether V-treated diabetic rats experience alterations in the metabolism of Fe and in the activity of liver enzymes related to antioxidant defence. The results obtained will clarify the role of V as a micronutrient and the level of its biological activity as an antidiabetic agent.

## 2. Materials and Methods

### 2.1. Animals and Diets

The experiment was carried out over a period of five weeks. Male *Wistar *rats (Charles River Laboratories, L' Arbresle, France) weighing 190–220 g at the beginning of the experimental period were randomly distributed into four groups: control group (C): 9 healthy rats; diabetic group (DM): 8 STZ-induced diabetic rats; diabetic treated group (DMV): 8 STZ-induced diabetic rats treated with 1 mg V/d (administered as 6.2 mg BMOV/d); and high-dose diabetic group (DMVH): 8 STZ-induced diabetic rats treated with 3 mg V/d (administered as 18.7 mg BMOV/d).

All groups were fed with the semisynthetic AIN-93 diet (which provided 44.5 mg Fe and 60 *μ*g V/kg food) and were allowed free access to drinking water (Milli-Q quality) throughout the experimental period. In the DM, DMV, and DMVH groups, type I diabetes was induced by single intraperitoneal administration of streptozotocin (STZ) (Sigma-Aldrich, St. Louis, MO, USA) at a dose of 60 mg/kg body weight, as a diabetogenic agent. Seven days later, the induction of diabetes was confirmed by measuring blood glucose levels on three occasions (9 a.m., 2 p.m., and 7 p.m.). Animals with a glucose concentration (random glycaemia) >13.8 mmol/L and with polyuria, polydipsia, and hyperphagia were considered diabetic [16].

In all cases, the BMOV solution was prepared and administered daily in the drinking water for 35 days, a sufficient time for the effects of V to become apparent [11, 17, 18]. BMOV was synthesised according to published procedures [19]. During the experimental period, the animals' weight gain and intake of food and water were monitored. Every day at 9 a.m., the surplus water from the previous day was removed and the amount consumed was calculated.  Table 1 shows the water and V intake.

On day 35, after overnight fasting, the rats were anaesthetised by intraperitoneal injection of 5 mg sodium pentobarbital/100 g body weight (Sigma-Aldrich, St. Louis, MO, USA) and exsanguinated by cannulating the posterior aorta. Blood was collected and centrifuged at 1200 ×g for 15 minutes to separate the serum. The gastrocnemius muscle, kidney, liver, spleen, heart, and femur were removed, weighed, placed in preweighed polyethylene vials, and stored at −80°C. During the last seven days of the experimental period, the faeces and urine were collected every 24 h and stored at −80°C in polyethylene bottles for subsequent analysis. All animals were housed from day 0 of the experiment in individual metabolic cages designed for the separate collection of faeces and urine. The cages were located in a well-ventilated, temperature-controlled room (21 ± 2°C) with relative humidity of 40–60% and a 12-hour light:dark period.

The following biological indices were calculated: absorbed as [I − F] and retained as [I − (F + U)], where I = intake, F = faecal excretion, and U = urinary excretion.

All experiments were carried out in accordance with Directional Guides Related to Animal Housing and Care (European Community Council, 1986) and all procedures were approved by the Animal Experimentation Ethics Committee of the University of Granada.

### 2.2. Analytical Methods

V and Fe in the diet, serum, and tissues were determined by ICP-MS (Agilent 7500, Tokyo, Japan) after digestion of the corresponding material using a microwave oven (Milestone, Sorisole, Italy). All the plastic containers used in the analysis were previously cleaned with superpure nitric acid and ultrapure water (18.2 *Ω*) obtained using a Milli-Q system (Millipore, Bedford, MA, USA). For Fe determination, 0.2 g of the corresponding samples was taken and 6 mL of a mixture (1 : 1) of nitric acid (65%) and hydrogen peroxide (30%), both of Suprapur quality (Merck, Darmstadt, Germany), was introduced directly into the microwaved vessels. The samples were then digested by applying the following programme: power 1000 W, 2 min ramp to 85°C, 4 min ramp to 135°C, 5 min ramp to 230°C held for 15 min. After digestion, the extracts were collected and made up to a final volume of 30 mL with Milli-Q water for subsequent analysis. Further dilutions of samples were prepared when necessary. Similarly, for the analysis of V, 0.5 g of each serum sample was treated with 8 mL of a mixture of nitric acid, hydrogen peroxide, and water in a 1 : 1 : 6 ratio in the microwave digester. After digestion, the extracts were collected and made up to a final volume of 10 mL with Milli-Q water for subsequent analysis. Calibration curves for Fe and V were prepared using stock solutions of 1000 mg/L of each element (Merck), using Ga as an internal standard. The internal standard was added to each sample in the same conditions. Calibration curves were prepared using 5 points with ranges of 0-1 mg/L and 0-0.1 mg/L for Fe and V, respectively. The total metal content in the tissues was obtained by interpolation in the corresponding graphs; for this purpose, each sample was measured three times. The accuracy of the method used was evaluated by comparison with certified reference materials such as Seronorm (Billingstad, Norway) and NIST 8414 (Gaithersburg, MD, USA) obtaining results of 68.9 ± 2.4 mg/kg for Fe and 0.00391 ± 0.00087 mg/kg for V, these values being in agreement with the certified ones (estimated in the case of V) of 71.2 ± 9.2 mg/kg and 0.0050 mg/kg, respectively. Additionally, spiking experiments were conducted: the concentration of Fe was analysed in three aliquots of the same sample, two of which had previously been enriched with different concentrations of Fe: 0.25 and 1 mg/L. The same procedure was carried out for V, using in this case concentrations of 0.06 and 0.1 mg/L. The recoveries calculated were 92–98% in all cases.

Glycaemia levels were determined using the sensor ACCU-CHEK AVIVA (Roche, Mannheim, Germany). Liver samples were homogenised in an ice-cold buffer (100 mM Tris-HCl, 0.1 mM EDTA, and 0.1% triton X-100 (v/v), pH 7.8) at a ratio of 1 : 9 (w/v). Homogenates were centrifuged at 30,000 rpm for 30 min in a Centrikon H-401 (Germany) centrifuge. After centrifugation, the supernatant was collected and frozen at −80°C until analysed. All enzymatic assays were carried out at 25 ± 0.5°C using a Power Wavex microplate scanning spectrophotometer (Bio-Tek Instruments, USA) in duplicate in 96-well microplates (UVStar, Greiner Bio-One, Germany). The enzymatic reactions were started by the addition of the tissue extract. The specific assay conditions were as follows.

Superoxide dismutase (SOD; EC 1.15.1.1) activity was measured spectrophotochemically by the ferricytochrome C method using xanthine/xanthine oxidase as the source of superoxide radicals [20]. The method was adapted according to Trenzado et al. [21]. The reaction mixture consisted of 50 mM potassium phosphate buffer (pH 7.8), 0.1 mM EDTA, 0.1 mM xanthine, 0.013 mM cytochrome C, and 0.024 IU mL^−1^ xanthine oxidase. One activity unit was defined as the amount of enzyme necessary to produce a 50% inhibition of the ferricytochrome C reduction rate measured at 550 nm.

Catalase (CAT; EC 1.11.1.6) activity was determined by measuring the decrease in H_2_O_2_ concentration at 240 nm [22]. The reaction mixture contained freshly prepared 10.6 mM H_2_O_2_ in 50 mM potassium phosphate buffer (pH 7.0).

NAD(P)H:quinone-oxidoreductase-1 (NQO1; EC 1.6.99.2) activity was determined by measuring DCPIP reduction at 600 nm [23]. The reaction mixture contained 50 mM Tris-HCl (pH 7.6), 50 *μ*M 2,6-dichlorophenol indophenol (DCPIP), and 0.5 mM NADH. A control reaction with distilled water (three measures average) was assayed and subtracted from each sample reaction. For these enzymes, one unit of activity is defined as the amount of enzyme required to transform 1 *μ*mol of substrate/min under the above assay conditions. The levels of protein carbonyl groups were assessed using Protein Carbonyl Kit (Cayman Chemical Company, MI, USA). The protein content of the supernatant solutions was determined by the Bradford method [24], using bovine serum albumin as the standard.

All biochemicals, including substrates, coenzymes, and purified enzymes, were obtained from Roche (Mannheim, Germany) or Sigma Chemical Co. (St. Louis, MO, USA). All other chemicals came from Merck (Darmstadt, Germany) and were of reagent grade.

### 2.3. Statistical Analysis

Descriptive statistical parameters (means and standard deviations) were obtained for each of the variables studied. Statistical comparisons among the four groups were performed by the analysis of the variance (ANOVA) test. For the bivariate analysis, Pearson's coefficient of correlation was calculated. All analyses were performed using the Statistical Package for Social Science 15.0 (SPSS, Chicago, IL, USA). Differences were considered statistically significant at a probability level <5%.

## 3. Results

Table 1 shows the food, water, and V intake and the digestive and metabolic utilisation of Fe. The diabetic rats showed an increase in food and water intake, in Fe excreted in faeces and urine, and in Fe absorbed, but no changes were found in Fe retained, compared to the nondiabetic control rats. Treatment with 1 mg V/d decreased the intake of food and water and the faecal excretion of Fe and increased Fe retained compared to those in the untreated diabetic rats. Treatment with 3 mg V/d decreased the intake of food and water, the faecal and urinary excretion of Fe, and Fe absorbed and retained compared to those in the diabetic untreated rats and to those in the diabetic rats treated with 1 mg V/d. The rats treated with 3 mg V/d had values of food and water intake, faecal excretion of Fe, and Fe absorbed and retained similar to those of the controls. However, treatment with 3 mg V/d provoked urinary losses of Fe greater than those recorded for the controls.

Table 2 shows the Fe content in the tissues studied. Diabetes provokes increased Fe levels in kidney, skeletal muscle, and femur and decreased Fe content in the spleen. Treatment with 1 mg V/d increased Fe levels in liver and femur, in comparison with those in the untreated diabetic rats. Treatment with 3 mg V/d restored Fe in the muscle to levels similar to those of the control group but increased the content of Fe in the liver, spleen, and heart versus that in the controls and untreated diabetic rats. Moreover, this dose increased Fe in spleen and heart, in comparison to that in the group treated with 1 mg V/d.

Table 3 shows the activities of SOD, CAT, NQO1, and protein carbonyl group levels. There was a decrease in CAT and an increase in NQO1 activity in the untreated diabetic rats. The two doses of V tested reduced the activity of NQO1 and increased that of CAT and protein carbonyl groups versus that in the controls and untreated diabetic rats.

The bivariate study revealed the existence of significant relations, among which the following are particularly important. V intake correlated negatively with Fe content in liver (*r* = 0.46; *P* < 0.01), heart (*r* = 0.43; *P* < 0.05), and femur (*r* = 0.55; *P* < 0.01). The intake of V also correlated positively with the activity of CAT (*r* = 0.61; *P* < 0.01) and protein carbonyl group levels (*r* = 0.46; *P* < 0.05).

## 4. Discussion

The doses of V used in the present study (1 mg V/d, approximately 5 mg V/kg body weight/d, and 3 mg V/d, approximately 15 mg V/kg body weight/d) are low in comparison with those administered in many other studies and much lower than LD50 (lethal dose 50) (41–90 mg V/kg body weight) [10, 11, 25]. The sample size has been designed according to most of the studies performed in this field [2, 10, 11, 13, 14].

The hyperphagia associated with diabetes leads to increased food (Fe) intake, which accounts for the increased net absorption of Fe observed in the untreated diabetic group [26]. Despite the high Fe absorption and high urinary losses of Fe, the polyuria that accompanies diabetes meant that the total amount of Fe retained in the untreated diabetic rats was similar to that of the controls (Table 1).

The treatment of diabetic rats with 1 mg V/d decreased food intake (Fe intake) in comparison with that in the untreated diabetic rats. It has been suggested that the decrease in food intake could be because V heightens the effects of leptin, thus reducing food intake [27]. Other authors have suggested that the reduction in food and water intake could be due to the fact that V reduces the increased production of neuropeptide Y (NPY) that occurs in diabetes [28]. However, in the treated animals the net quantity of Fe retained increased (Table 1). In this group, there was a reduction in faecal losses of the cation, which would account for the increased amount of Fe retained, in comparison with that in the untreated diabetic rats.

The dose of 3 mg V/d administered to the diabetic rats had a clear antidiabetic effect, normalizing glycaemia [12] and reducing the hyperphagia (Fe intake) and polydipsia that accompany diabetes; food and water intake approached the levels found in the controls (Table 1). The reduced food consumption was the main reason why the rats in this group presented values of Fe absorbed similar to those of the controls and significantly lower than those of the other groups of diabetic rats.

In the group of untreated diabetic rats, the Fe content increased in various tissues. Previous studies have also described increased Fe content in various organs, and in this respect there is a high degree of agreement in the case of the kidney [14, 15]. One study showed that renal transferrin receptor expression increased in STZ-induced diabetic rats [13], which could explain the accumulation of Fe observed in the kidney (Table 2). Treatment with 3 mg V/d produced a significant increase in Fe content in the liver, heart, and spleen (Table 2), possibly due to the erythropoietic suppression and the greater uptake of Fe by hepatocytes, macrophages, and cardiac myocytes. The increased Fe content in the heart reveals the existence of Fe overload, because the heart is an organ where Fe is deposited when the storage organs become saturated. This overhead indicates a worsening of the situation, which could cause malfunctions in the organs affected.

The presence of metal ions (especially Fe) and the activation of macrophages by cytokines and/or abnormal metabolites are known to lead to an overproduction of reactive oxygen species (ROS). To determine whether the increased Fe in the liver affects the oxidative status of this organ, we designed a study of the activity of enzymes (SOD, CAT) directly related to the control of the iron-induced ROS production (Table 3). In addition, NQO1 activity was studied (Table 3). The activity of NQO1 increases in response to xenobiotics, oxidants, heavy metals, and carcinogens, which has led to the suggestion that the enzyme may offer protection against the toxic effects of these agents [29]. In order to determine whether the generation of ROS caused protein oxidative damage, we examined protein carbonyl group levels. These abnormal metabolites, too, favor the production of ROS. The liver was chosen for this study due to its importance in Fe metabolism and because the treatment produced significant Fe deposits in this organ.

Our results showed that in the untreated diabetic rats there were no changes in the activity of SOD, while CAT decreased and NQO1 increased. These findings are consistent with those of previous studies concerning SOD [30, 31] and CAT [32]. The increased NQO1 activity in diabetic rats may favor a decrease in the formation of reactive oxygen species, which would explain why no changes in SOD appear, together with the decreased CAT activity in this group. Treatment with 1 mg V/d increased the hepatic content of Fe, which was accompanied by nonsignificant increases in SOD, increased hepatic CAT, and raised levels of protein carbonyl groups. Treatment with 3 mg V/d caused an increase in Fe content in the tissues. Fe was deposited mainly in the kidney, liver, spleen, and heart. This situation was accompanied by an increase in CAT activity and a decrease in that of NQO1 in the liver. Recent *in vitro *studies with HepG2 cells [33] have shown that exposure to V (ammonium metavanadate) at low concentrations does not modify the activity of NQO1 but that the latter decreases when the dose is increased. These authors suggested that V inhibits the gene expression of NQO1.

Although our study used a different V compound and our results were obtained *in vivo*, they corroborated those mentioned above; with a dose of 1 mg V/d, no significant changes were observed, whereas enzyme activity decreased when the dose of 3 mg V/d was used, in relation to that in the untreated diabetic rats. Previous studies have shown that V decreases GPx activity and increases malondialdehyde levels in the livers of healthy rats [34] and of diabetic rats treated with V [12]. The decrease in NQO1 and GPx activity, together with the enlarged pool of liver Fe, may have increased the production of hydroxyl radicals. The strong CAT activity response appears to be insufficient to prevent the increased formation of protein carbonyl groups, which would suggest that the treatment provoked oxidative damage to the liver proteins.

## 5. Conclusion

Our results showed that treatment with 3 mV/d (administered as 18.7 mg BMOV/d) caused an iron overload in tissues that impaired antioxidant defences and produced protein oxidative damage in the liver. However, further studies are needed to better determine the effects arising from these interactions in order to determine optimum levels for pharmacological use, thus reducing or preventing toxic effects.

## Figures and Tables

**Table 1:** Digestive and metabolic utilization of Fe on days 28–35 of study.

	C	DM	DMV	DMVH	*P*_test_
Food intake (g/day)	15 ± 2	33 ± 2.4^a^	26.9 ± 2^a,b^	13.8 ± 1.1^b,c^	*P* < 0.001
Water intake (mL/day)	17 ± 4	324 ± 36^a^	191 ± 41^a,b^	14 ± 7^b,c^	*P* < 0.001
V intake (*µ*g/day)	1 ± 0.1	1.9 ± 0.1^a^	965 ± 104^a,b^	3228 ± 350^a,b,c^	*P* < 0.001
Fe intake (*I*) (*µ*g/day)	668 ± 88	1472 ± 105^a^	1197 ± 89^a,b^	614 ± 50^b,c^	*P* < 0.001
Faecal excretion (*F*) (*µ*g/day)	497 ± 75	1219 ± 106^a^	888 ± 50^a,b^	430 ± 78^b,c^	*P* < 0.001
Urinary excretion (*U*) (*µ*g/day)	29 ± 8	118 ± 95^a^	103 ± 22^a^	47 ± 17^a,b,c^	*P* < 0.001
Absorbed (*A*); *A* = (*I* − *F*) (*µ*g/day)	171 ± 78	253 ± 56^a^	308 ± 68^a^	184 ± 59^b,c^	*P* < 0.01
Retained (*R*); *R* = [*I* − (*F* + *U*)] (*µ*g/day)	142 ± 79	134 ± 52	205 ± 66^b^	137 ± 64^c^	NS

Values shown are means ± SD, C: control rats; DM: diabetic STZ rats; DMV: diabetic STZ rats treated with 1 mgV/day; DMVH: diabetic STZ rats treated with 3 mgV/day. ^a^Different from group C; ^b^different from group DM; ^c^different from group DMV. *P* < 0.05. NS: not significant.

**Table 2:** Iron content in kidney, liver, spleen, muscle, heart, and femur (mg/kg dry tissue) on day 35.

	C	DM	DMV	DMVH	*P*_test_
Kidney	255 ± 43	359 ± 74^a^	299 ± 51	290 ± 53	*P* < 0.05
Liver	404 ± 54	428 ± 65	505 ± 82^a,b^	530 ± 73^a,b^	*P* < 0.05
Spleen	3043 ± 646	1849 ± 718^a^	2410 ± 670	3918 ± 671^a,b,c^	*P* < 0.001
Muscle	50 ± 14	68 ± 19^a^	70 ± 14^ a^	46 ± 5^b,c^	*P* < 0.01
Heart	340 ± 26	353 ± 56	356 ± 44	420 ± 46^a,b,c^	*P* < 0.05
Femur	40 ± 4	61 ± 4^a^	69 ± 6^a,b^	64 ± 19^a^	*P* < 0.001

Values shown are means ± SD, C: control rats; DM: diabetic STZ rats; DMV: diabetic STZ rats treated with 1 mgV/day; DMVH: diabetic STZ rats treated with 3 mgV/day. ^a^Different from group C; ^b^different from group DM; ^c^different from group DMV. *P* < 0.05.

**Table 3:** Activity of superoxide dismutase (SOD), catalase (CAT), NAD(P)H: quinone-oxidoreductase-1 (NQO1), and protein carbonyl group levels in the liver on day 35.

	C	DM	DMV	DMVH	*P*_test_
SOD (U/mg protein)	685 ± 81	672 ± 83	743 ± 74	718 ± 70	NS
CAT (U/mg protein)	389 ± 97	295 ± 29^a^	402 ± 76^b^	733 ± 73^a,b,c^	*P* < 0.001
NQO1 (mU/mg protein)	173 ± 72	269 ± 87^a^	242 ± 59	155 ± 57^b,c^	*P* < 0.05
Protein carbonyl groups (pm/mg protein)	134 ± 33	130 ± 38	196 ± 40^a,b^	186 ± 21^a,b^	*P* < 0.01

Values shown are means ± SD. C: control rats; DM: diabetic STZ rats; DMV: diabetic STZ rats treated with 1 mgV/day; DMVH: diabetic STZ rats treated with 3 mgV/day. ^ a^Different from group C; ^b^different from group DM; ^c^different from group DMV. *P* < 0.05. NS: not significant.
